# Ricin: An Ancient Story for a Timeless Plant Toxin

**DOI:** 10.3390/toxins11060324

**Published:** 2019-06-06

**Authors:** Letizia Polito, Massimo Bortolotti, Maria Giulia Battelli, Giulia Calafato, Andrea Bolognesi

**Affiliations:** Department of Experimental, Diagnostic and Specialty Medicine—DIMES, General Pathology Section, Alma Mater Studiorum—University of Bologna, Via S. Giacomo 14, 40126 Bologna, Italy; massimo.bortolotti2@unibo.it (M.B.); mariagiulia.battelli@unibo.it (M.G.B.); giulia.calafato2@unibo.it (G.C.)

**Keywords:** bioterrorism, cancer therapy, castor bean, folk medicine, immunotoxins, plant toxins, ribosome-inactivating proteins, ricin, rRNA N-glycosylase activity, traditional medicine

## Abstract

The castor plant (*Ricinus communis* L.) has been known since time immemorial in traditional medicine in the pharmacopeia of Mediterranean and eastern ancient cultures. Moreover, it is still used in folk medicine worldwide. Castor bean has been mainly recommended as anti-inflammatory, anthelmintic, anti-bacterial, laxative, abortifacient, for wounds, ulcers, and many other indications. Many cases of human intoxication occurred accidentally or voluntarily with the ingestion of castor seeds or derivatives. Ricinus toxicity depends on several molecules, among them the most important is ricin, a protein belonging to the family of ribosome-inactivating proteins. Ricin is the most studied of this category of proteins and it is also known to the general public, having been used for several biocrimes. This manuscript intends to give the reader an overview of ricin, focusing on the historical path to the current knowledge on this protein. The main steps of ricin research are here reported, with particular regard to its enzymatic activity, structure, and cytotoxicity. Moreover, we discuss ricin toxicity for animals and humans, as well as the relation between bioterrorism and ricin and its impact on environmental toxicity. Ricin has also been used to develop immunotoxins for the elimination of unwanted cells, mainly cancer cells; some of these immunoconjugates gave promising results in clinical trials but also showed critical limitation.

## 1. Castor Bean in Traditional and Folk Medicine

Ricin derives from *Ricinus communis* L. (Euphorbiaceae family), also known as castor bean or *palma Christi*. The genus Ricinus has only one known species: the castor oil plant. The plant possibly originates from Africa and Asia and now is widespread throughout temperate, subtropical, and tropical areas, growing as an invasive plant or being cultivated for different purposes.

The castor plant has been known since time immemorial and its use in the prehistoric era has been evidenced by archaeological findings such as that of the Border Cave in South Africa. Traces of wax containing ricinoleic and ricinelaidic acids were found on a thin wooden stick, which was suggested to be a poison applicator, dating back to about 24,000 years ago [1]. The castor seeds and other parts of the castor plant were certainly utilized in ancient Egypt for pharmacological purposes. In the Ebers Papyrus, an Egyptian medical treatise dating back to before 1500 BCE, an entire chapter is dedicated to the castor bean that is indicated as an abortifacient, a laxative, a remedy for abscessual illness, baldness, and so on [2]. In the Hearst Papyrus, written approximately in the same period, various castor plant parts are included as ingredients in some prescriptions for internal use, with the aim of expelling fluid accumulation or promoting diuresis, as well as for external use as poultices for bandaging [3]. Ancient Egyptians knew the toxicity of castor bean and the use of seed pulp, included in drug preparations for oral ingestion, was recommended only in small amounts. In addition, a castor seed-containing concoction was prescribed to cure the urinary disease of a possibly diabetic child [4]. Around 400 BCE, the father of western medicine Hippocrates prescribed castor bean oil for laxative and detoxifying action [5]. The Greek herbalist and physician Pedanius Dioscorides (40 to 90 CE) in *De Materia Medica* wrote that castor seeds could be used as expectorant, diuretic, emetic, laxative, anti-inflammatory, to cure erysipelas, burns, varicose veins, etc. [6]. In the same period, Pliny the Elder (23 to 79 CE) wrote *Naturalis historia*, comprising the whole area of antique knowledge. In this encyclopedic work, also castor bean found a place [7].

Castor bean was used also in the pharmacopeia of eastern ancient cultures. In Chinese traditional medicine, castor seeds were recommended for their anthelmintic activity; seed poultice and leaf juice were prescribed for external use to treat ulcers and chronic wounds, whereas the latex was instilled in the ear for rhinitis treatment (reviewed in [8]). In Ayurveda, castor plant is used for rheumatic conditions, as well as for gastropathy, constipation, inflammation, fever, ascites, bronchitis, cough, skin diseases, colic, and lumbago. In Yunani medicine, castor root is used as a purgative and for skin diseases, the leaves are used to increase breastmilk production and are applied to skin for burns, the seeds and the oil act as a purgative, useful in liver troubles, pains, lumbago, boils, piles, ringworm, inflammation, ascites, asthma, rheumatism, dropsy, and amenorrhea (reviewed in [9]). Ground castor seeds or leaf paste have been applied in veterinary medicine to heal sprains, swelling, and wounds [10].

Castor bean has been used in folk medicine throughout the world and has been reported: (i) As a galactogogue on the Mediterranean coasts of Europe, where fresh leaves or leaf juice are applied on the puerperal breast to promote lactation; (ii) as a remedy for various articular, cutaneous, or ocular diseases in Africa, where crushed seeds or oil, sometimes in combination with other plants, are spread or rubbed on the part of the body in need, or a root decoction is drunk to induce uterine contraction as an abortive; (iii) as a medicament to cure erysipelas, flu, inflammation of the womb, and stomach aches in the Caribbean, where a leaf poultice is recommended; (iv) as an anthelmintic or a purgative in Brazil where the seed oil is orally consumed, or locally applied with the purpose of stopping hair loss, healing wounds, or burns (reviewed in [11]).

The laxative and abortifacient activities of castor seeds have been attributed to the activation of intestinal and uterine smooth-muscle cells via prostaglandin EP3 receptors induced by ricinoleic acid [12]. Castor oil-induced diarrhea can be antagonized by hexane extract of *Citrus limon* peel that activates antisecretory and antimotility mechanisms through the β adrenergic system [13]. The purgative and anthelmintic actions of the oral ingestion of castor seeds, at least in part, have been ascribed to the irritating effect caused to the intestine by ricin, as reported in toxicological studies (reviewed in [14]). In addition, the antiflogistic action of castor bean could be related to the high toxicity of ricin to macrophagic cells, which are responsible for producing inflammatory cytokines (reviewed in [15]). This effect, together with the anti-pathogen activity of ricin, could promote healing of the lesions, thus justifying its use in the treatment of various skin conditions.

## 2. The Ricin Story

Castor seed toxicity began to be investigated at the end of nineteenth century at Schmiedeberg’s laboratory in Strasbourg. The toxic component of Ricinus could be extracted with water and precipitated with alcohol, but it lost its toxic activity through heating, treatment with strong acid, or repeated precipitation with alcohol. In 1887, Dixson supposed that the toxicity of Ricinus was due to an albumen-like toxic body [16]. However, it was still unknown whether the seed toxicity was due to a protein or a glycoside (reviewed in [17]). The problem was solved at the Medical Faculty of Dorpat (now Tartu) where an extremely toxic protein was partially purified from castor seed or press cake and named ricin. This finding was published in the doctoral thesis written by Hermann Stillmark under the supervision of Prof. Rudolf Kobert [18]. Stillmark noticed the agglutinating activity of ricin on red blood cells, that had mistakenly been believed to be the cause of ricin toxicity until the agglutinin was separated from the toxin [19]. 

Paul Ehrlich began his experiments in immunology by feeding mice with small amounts of ricin or abrin, another similar plant toxin, until they were accustomed and became resistant to the toxin used, yet still remaining sensitive to the other toxin. The immunization was strictly specific, started after a few days, and persisted at least for several months [20,21]. He was successful in the production of antisera against abrin and ricin and in the determination of antibody titer in serum and milk. Ehrlich drew animal experiments that clarified the transmission of passive immunity from mother to offspring through the transplacental transfer of antibodies and through breastfeeding. He investigated the dynamics of the antibody response and was the first to envisage the presence of binding sites on the cell surface (reviewed in [22]). These studies, together with those on the immunity to bacterial toxins, led him to formulate his side-chain theory of antibody formation and to win, in 1908, the Nobel Prize [23].

**Figure 1:**
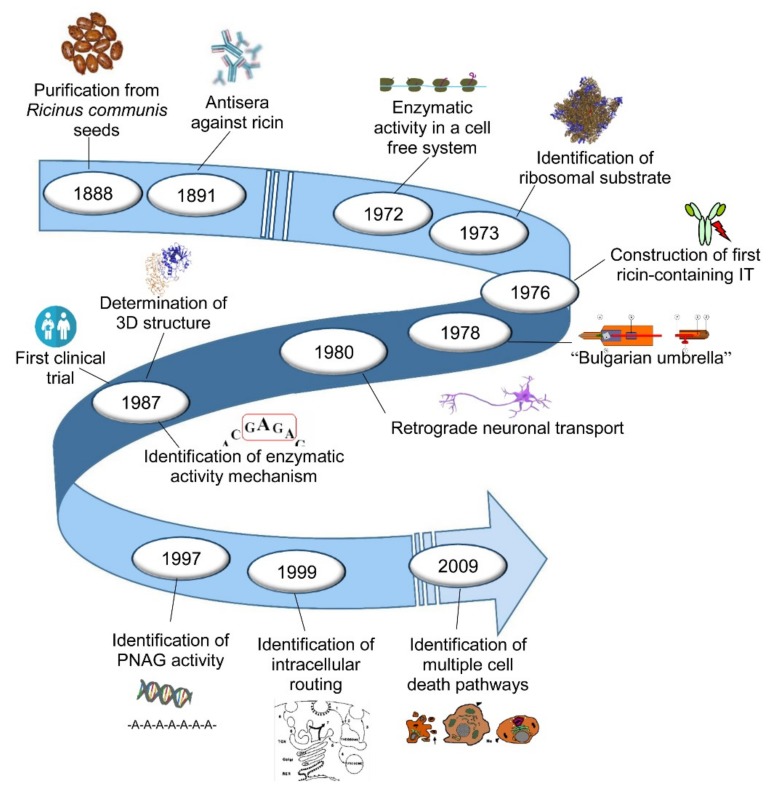
The main milestones of ricin research.

Interest in ricin was rekindled when the anticancer activity of this toxin on Ehrlich ascites cells in a mouse model was published [24]. A strong inhibition of protein synthesis by ricin was observed in cultures of both Ehrlich ascites tumour cells and Yoshida ascites hepatoma cells. The inhibition of protein synthesis by ricin requires more time in rat liver than in neoplastic cells [25]. The prospect of a possible use in cancer therapy highlighted the need to investigate which part of the proteosynthetic machinery was damaged and how the toxin managed to enter the cell to reach its target. Hereinafter, we highlight the milestones of research on ricin, with particular regard to its enzymatic activity, structure, cytotoxicity, toxicity for animals and humans, and its use as an immunotoxin, used in experimental models and in clinical trials. The main milestones are shown in Figure 1.

### 2.1. Ricin Structure

**Figure 2:**
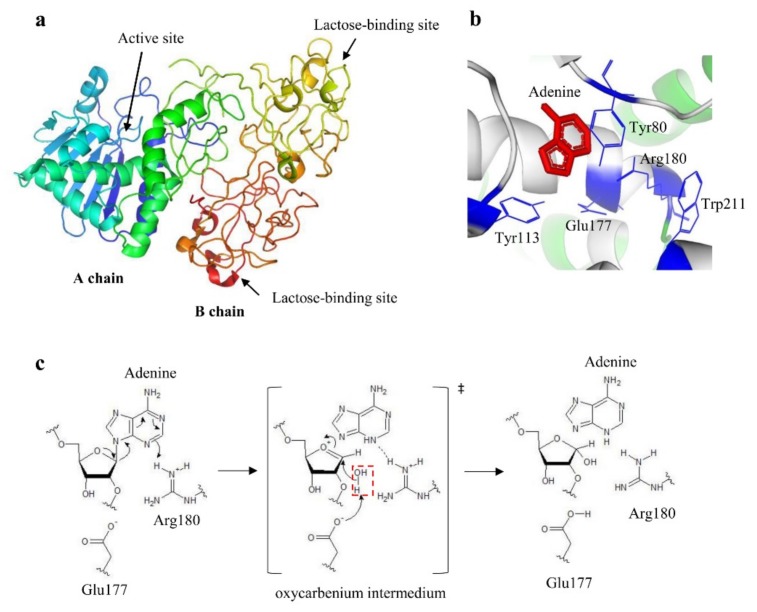
(**a**) Ribbon model of the crystal structure of ricin at 2.5 Å (accession number Protein Data Bank 2AAI). The A chain domains are colored in green, blue, and light blue; the B chain domains are colored in yellow and orange. (**b**) Catalytic site of ricin. The key residues are indicated and colored in blue, whereas adenine substrate is depicted in red. (**c**) Proposed mechanism of depurination reaction catalyzed by ricin. The hydrolysis proceeds through a dissociative mechanism forming an oxocarbenium transition state. Arg180 protonates the leaving group and the N-glycosidic bond is broken. Glu177 deprotonates the hydrolytic water (highlighted by a red dotted rectangle) that attacks carbon to complete the depurination reaction. Figure 2a and 2b were produced by PyMOL (version 2.3.1); Figure 2c was produced by ACD/ChemSketch (version 2015.2.5).

The first information about the bi-chain nature of ricin structure dates to the early 1970s, when it was shown that ricin was composed of two chains, A (active) and B (binding), linked together through a disulphide bond [26,27]. In the same period, the complete primary sequence of the ricin A and B chains was determined [28,29]. Ricin holotoxin structure was solved for the first time at 2.8 Å resolution (Figure 1) [30]. This pioneering work demonstrated that ricin A chain was a globular protein folded into three domains all contributing to the active site, while the B chain lectin folded into two domains, each binding lactose in a shallow cleft. The interface between the A and B chains showed some hydrophobic contact in which proline and phenylalanine side chains played a prominent role. Four years later, the same researchers refined ricin structure at 2.5 Å (Figure 2a), allowing a more detailed molecular description of the holotoxin and of the separated A and B chains [31,32,33]. Ricin A chain has been described as a globular protein consisting of 267 amino acids and organized in 8 α-helices and 8 β-strand structures. Ricin B chain consists of 262 amino acids and two homologues domains, each containing a lactose binding site and several areas of amino acid homology, possibly derived from a gene duplication. In 1995, after purification of a complex of ricin A chain cross-linked to the ribosome, it was found the binding of ricin A chain with the ribosomal proteins L9 and L10e [34,35].

The knowledge of the tridimensional structure of ricin yielded more information on its active site. Studies based on the formation of complexes between the A chain, both native and recombinant, and adenine-containing nucleotides allowed for the identification of key residues in enzymatic activity. In particular, Tyr80, Tyr123, Glu177, Arg180, and Trp211 were found to form the binding site for adenine (Figure 2b) [30,36]. In the 1990s, the molecular mechanism of adenine release was hypothesized: Adenine is sandwiched between Tyr80 and Tyr123 in a π stacking interaction; the N3 of adenine is protonated by Arg180, promoting the C1’-N9 bond breaking, and thus forming an oxocarbenium moiety on the ribose (Figure 2c) [36,37]. This transition state is stabilized by Glu177; a water molecule lies on the opposite side of the sugar ring from adenosine, which will be polarized by Arg180 to a hydroxide character that rapidly attacks the sugar carbon completing the reaction.

### 2.2. Ricin Enzymatic Activity 

The introduction of a cell-free system utilizing a lysate from rabbit reticulocytes [38] helped to clarify that ricin inhibited the peptide chain elongation (Figure 1) [27]. The two polypeptides showed different properties: The A chain possessed the toxic activity, while the B chain was a galactose-specific lectin binding the cell surface [26]. Treating the toxin with reducing agents resulted in more activity in inhibiting cell-free protein synthesis [39]. Firstly, the target of the toxic action was identified as the ribosome (Figure 1), then as the 60 S subunit of eukaryotic ribosome [40], which became unreactive toward elongation factors [41]. The toxin was found to prevent the binding between elongation factors and ribosomes avoiding the subsequent elongation-factor-dependent GTPase activity [41,42]. The A-chain molecule was very active on its substrate and it was calculated that one molecule can inactivate 2000 ribosomes/min, with a K_m_ of 0.1–0.2 mM [43].

In addition to ricin, several other plant proteins have been identified to possess a similar protein synthesis inhibiting action. Most of them had a single polypeptide chain similar to the A chain of ricin. They were called Ribosome-Inactivating Proteins (RIPs) (reviewed in [44,45]).

The already supposed enzymatic nature of ricin A chain was finally demonstrated in 1987 by Endo et al. who discovered that ricin A-chain cleaved the N-glycosidic bond of an adenine residue, A4324 in rat 28 S RNA, from the ribose of a highly conserved ribosomal RNA single-stranded loop involved in the binding of elongation factors (Figure 1). The toxin did not directly break the RNA chain, but the depurinated RNA was susceptible to hydrolysis [46,47]. Consequently, ricin activity was identified as an rRNA N-glycosidase (EC 3.2.2.22). 

Following this, it was demonstrated that the enzymatic activity of RIPs was broader than previously described. All tested RIPs were able to release adenine from DNA, in addition to rRNA, and some of them were also able to act on other polynucleotide substrates, releasing adenine from the sugar phosphate backbone of polynucleotide substrates (Figure 1) [48,49]. For this reason, the name of adenine polynucleotide glycosylase was proposed for RIPs. Thus, the ability of acting on various substrates and extensively depurinating some of them, suggested that the protein synthesis inhibition could be only one of the ways of RIP-mediated cell killing. Ricin was shown to be able to release adenine from rRNA, DNA (chromatin and naked), and also poly(ADP-ribosyl)ated poly(ADP-ribose) polymerase, an enzyme involved in DNA repair [48,50]. Furthermore, it was observed that many RIPs were able to cleave more than one adenine: ricin was able to detach few adenines from the DNA (tens), but some single-chain RIPs were able to detach even thousands of them. The hypothesis that ricin could act directly on DNA in cellular models was strengthened by the evidence that damage to nuclear DNA, consistent with the enzymatic activity (adenine release) on DNA in cell-free systems, was concomitant with protein synthesis inhibition and preceded apoptosis [51].

### 2.3. Ricin Cellular Uptake, Routing, and Toxicity

Starting from the mid-1970s, several research groups focused on ricin binding and internalization studies, demonstrating that the interaction of ricin with the cell started from the binding of the B chain to galactosyl residues on the cell surface, allowing access to the endosomal compartment [52]. Ricin binds to both glycolipids and glycoproteins with terminal galactose. Since ricin binds to a variety of different molecules, it seems to be internalized by different endocytic pathways, as well as by using different pathways to reach the Golgi apparatus to intoxicate the cell. In HeLa cells, about 10^7^ binding sites were found for ricin, but only small amount of the bound toxin reached the Golgi network and participated in cell intoxication [52]. 

Firstly, it was reported that ricin entered into cytoplasm through clathrin-dependent endocytosis [53]. Afterwards, it became clear that clathrin-independent mechanisms were also involved [54]. After cell uptake, ricin is delivered to early endosomes, from where most of protein molecules are recycled back to the cell surface or delivered, via late endosomes, to lysosomes for proteolytical degradation. A small amount of non-degraded ricin is addressed within the trans-Golgi network [55]. The involvement of the Golgi complex in ricin routing was confirmed using different Golgi-disrupting agents, such as brefeldin A, monensin, etc. In fact, the pretreatment with these agents inhibited the cytotoxic effects of ricin [56]. It was demonstrated that ricin was cycled from Golgi to the endoplasmic reticulum via coatomer protein 1 (COP-1)-coated vesicles [57], although it was later proved that the COP-1-independent pathway could also be involved [58]. 

The complete elucidation of intracellular ricin traffic occurred when it was demonstrated that, after reaching the endoplasmic reticulum, the two ricin chains were separated, and the A chain was retro-translocated through the quality control pathway delivering misfolded proteins to cytosol (Figure 1) [59]. Recently, it has been demonstrated that cholesterol rafts are required for Golgi transport of ricin; meaning that glycosphingolipids may not be required (reviewed in [60]). 

The portion of A chain that quickly refolded, thus avoiding ubiquitination and proteosomal degradation, was able to reach its intracellular target (reviewed in [61]). It was estimated that one molecule of active ricin that arrives to its substrate is enough to kill one cell [62]. 

The discovery that ricin, and some related toxins, may be retrogradely transported along neuronal processes (Figure 1) [63] opened a new field of research in neurobiology and this property has been exploited for the selective destruction of neuron bodies. 

Different cell types have shown variable levels of sensitivity to ricin (reviewed in [14]), possibly because of the mannose receptor expression on the cell surface and endocytosis efficacy. Ricin has been shown to be one of the most toxic plant toxins on cell lines with IC_50_s (concentration inhibiting protein synthesis by 50%) ranging from less than 0.1 to 1 pM [26,64,65,66]. However, it must be taken into account that it is very difficult to make a direct comparison of the data available in the literature about ricin cytotoxicity, because of the differences in the experimental approaches and technical conditions.

The polynucleotide depurinating activity of RIPs suggests the possibility of a wider toxic action on many biological substrates, not excluding the induction of oxidative stress. This could explain the induction of more than one cell death pathway, e.g. apoptosis and necroptosis, caused by ricin and other RIPs (Figure 1) [64,67].

## 3. Ricin Toxicity in Humans and Animals

On one hand, ricin has been studied for bio-medical applications, exploiting the ability of the A-chain to kill target cells once linked to a monoclonal antibody, as below described in the immunotoxins chapter. On the other hand, ricin has attracted nefarious interests, with a history of military, criminal, and terroristic uses [68].

The acute toxicity of ricin is highly variable depending on the animal species and strain. The pathological effects and subsequent clinical signs of ricin intoxication depend also on the route of exposure, as this dictates the subsequent tissue distribution of the toxin. Following intravenous or intramuscular administration, lesions eventually develop in the spleen, liver, and kidney whilst the lung remains unaffected. After oral ingestion, the gastrointestinal tract is severely affected. Inhalational exposure produces effects that are mainly confined to the respiratory tract [69].

The majority of data on animal toxicity has been derived from laboratory experiments in rodents, principally rat and mouse models. Oral administration of ricin was reported to give a lethal dose (LD) for 50% of animals (LD_50_s) 20 to 30 mg/kg in rat and 15 to 35 mg/kg in mouse [70,71,72]. For intravenous, inhalation and intraperitoneal routes, toxicity is approximately 1000-fold higher than that obtained for the oral route, with LD_50_ values in mouse of 2 to 10 µg/kg, 3–5 µg/kg and 22 µg/kg, respectively [70,73]. The lower toxicity of ricin after oral exposure is due to the protein destruction in the lumen of the intestinal tract [74,75]. Ricin acts in a time- and concentration-dependent manner. Notably, there is a time delay of about 10 h before death occurs, even when very high doses are applied [76].

### 3.1. Oral Toxicity 

In humans, most intoxications occurred accidentally or voluntarily with the ingestion of castor seeds; only a few cases of intentional absorption of castor bean extracts have been documented in suicide attempts [76]. Whole-ingested beans can pass intact through the gastrointestinal tract, whereas chewing facilitates ricin release. Also, it has been reported that the seed can act as ‘timed-release’ capsule for the toxin, allowing its release in the lower bowel, where it causes more damage [72]. After ingestion, vomiting, diarrhea, and abdominal pain are common symptoms. Massive gastrointestinal fluid and electrolyte loss are described, often complicated by hematemesis or melaena. Finally, hypovolemic shock and multiorgan failure occur, which particularly involves the spleen, liver, and kidney [77,78].

Despite the high number of intoxicated subjects with castor beans, it is quite difficult to calculate LD values for ricin in humans. In fact, the effective ingested ricin dose can only be supposed, because of ricin content variations depending on the size, weight, and moisture of the seeds, as well as on cultivar, region, season, and plant growth stage. Moreover, in intoxicated subjects, it must be taken into account the degree of mastication, stomach content, age, and comorbidities, parameters that are obviously more heterogeneous compared to experimental poisoning of animals. Considering all these parameters, the fatal oral dose of ricin in humans has been estimated to range from 1 to 20 mg/kg (approximately 5 to 10 beans) [70,79].

### 3.2. Inhalation Toxicity 

No data are available for human ricin uptake by inhalation. In non-human primates, LD_50_ has been estimated to be 5 to 15 μg/kg depending on aerosol particle size. Inhalation of particles that are able to penetrate deeply into the lungs (1 to 5 μm diameter) display much more toxicity than larger particles [72,80]. Inhalation of ricin causes slow onset of respiratory distress (difficulty breathing), coughing, fever, pulmonary lesions, and edema. Intoxicated animals develop fibrinopurulent necrotizing pneumonia accompanied by necrotizing lymphadenitis, typically after a dose-dependent delay of 8 to 24 h. Death occurs as a result of respiratory failure due to massive alveolar fluid accumulation. The liver, kidney, and small intestines appear congested, although little histologic changes have been shown [72,80,81].

### 3.3. Parenteral Toxicity 

Data regarding parenteral ricin intoxication derive mainly from animal studies. By injection, mice had an LD_50_ of 3 to 5 µg/kg by intravenous and 22 µg/kg by subcutaneous route [82], rabbits had LD_50_ 0.5 μg/kg by the intravenous route and 0.1 μg/kg by the intramuscular route, while guinea pigs had LD_50_ <1.1 μg/kg by the intravenous route and 0.8 μg/kg by the intramuscular route [83]. Human data only derives from the few cases of suicide or murder, or their attempt; the most known episode is the assassination of the Bulgarian dissident Georgi Markov, who in 1978 died three days after possibly being stabbed with an umbrella loaded with a ricin-containing pellet (Figure 1) [84].

By parenteral administration, immediate local pain at the injection site is reported, followed by general weakness within five hours. The following symptoms, that are general and maybe similar to sepsis (fever, headache, dizziness, anorexia, nausea, vomiting, hypotension, abdominal pain), can be delayed for as much as 10 to 12 h, even with high doses. Usually local tissue damage at the site of the injection is observed. Laboratory abnormalities included elevated liver transaminases, amylase and creatinine kinase, hyperbilirubinemia, myoglobinuria, and renal impairment. The clinical course may progress to multisystem organ failure. Preterminal complications included gastrointestinal hemorrhage, hypovolemic shock, and renal insufficiency [78,84].

## 4. Bioterrorism and Environmental Toxicity 

Ricin is currently monitored as Schedule 1A of the Chemical Weapons Convention (CWC) and is a Category B substance under the Biological and Toxins Weapons Convention (BTWC) [80]. Despite its toxicity, ricin is less potent than other agents, such as botulinum neurotoxin or anthrax. It has been estimated that eight tons of ricin would have to be aerosolized over a 100 km^2^ area to achieve about 50% casualty, whereas only a kilogram of anthrax spores would cause the same effect [85]. Thus, deploying an agent such as ricin over a wide area, although possible, becomes impractical from a logistics standpoint. However, the availability of castor beans and the quite simple procedure for rough ricin purification have attracted criminal and terrorist interest for small scale biocrimes or to cause collective media-driven alarm [80].

From castor seeds, a nontoxic oil can be extracted that has multitude of uses in many sectors, including cosmetic, pharmaceutic, mechanical, and chemical industry. Castor oil production is increasing worldwide because of its versatile application, low cost, availability, and biodegradability. In addition, the oil-free seed pulp can be used in agriculture as a natural fertilizer [86], although the processing of castor seeds requires great caution due to the high allergenicity [87,88] and extreme toxicity [76] of their protein fraction, represented, above all, by ricin. World production of castor oil increased from 0.8 million tons in 2000 [89] to 1.21 million tons in 2014 [90], with a castor seed production of 1.49 million tons in 2017 [91]. Leading producing countries are India, with over 80% of the global yield, Mozambique, China, Brazil, Myanmar, Ethiopia, Paraguay, and Vietnam [92]. The oil makes up about 50% of the weight of the seeds and is mostly constituted of ricinoleic acid (90%), with minor amounts of dihydroxystearic, linoleic, oleic, and stearic acids. Ricin isoforms and the alkaloid ricinine, are not transferred to the oil fraction during extraction, which can be performed by cold or warm pressing, but remain in the seed cake [93,94].

Castor bean meal press cake or other residues of the castor oil production have been employed as a protein source for feed or fertilizer, but their use is very limited due to ricin toxicity [76]. In 2008, the European Food Safety Agency defined ricin as an undesirable substance in animal feed. Ricinus derived material should be appropriately inactivated through physical and/or chemical methods to guarantee animal and human health [95]. Nevertheless, many accidental poisonings are still reported for animals eating improperly detoxified fertilizer or other agricultural products containing castor derived material [76,94]. 

In order to block the toxic action of ricin, different strategies have been evaluated: Vaccines, inhibitors, and passive immunity. Vaccines against ricin with the consequent production of neutralizing antibodies did not give satisfactory results in vivo (reviewed in [96]). Inhibitors of ricin can block the active site or work as a substrate analogue; however, the available data are limited to in vitro experiments [97]. More recently, inhibitors of cell routing have been developed, sometimes giving promising results, also in vivo [98,99]. To date, passive immunity has proven to be the only effective strategy for treating intoxication caused by ricin. The delay in the appearance of signs of intoxication makes confirmation of exposure, diagnosis of intoxication, and the subsequent medical response technically and logistically challenging. The development of anti-ricin sera or antibodies, effective even when used several hours after toxin exposure, represents a step forward in treatment of ricin intoxication, as it increases the time window of intervention (WOO, window of opportunity). Many authors described effective post-exposure treatment of ricin intoxication with specific antibodies, but with a limited WOO (~8 h) [100,101,102,103]. Other authors reported a survival between 50% and 89% of mice treated with anti-sera 24 h after intoxication [104,105,106]. Once internalized into the cells, ricin cannot be neutralized by antibodies, thus limiting the therapeutic window. However, Whitfield et al. in 2017 reported 100% protection in aerosolized ricin-treated mice with a single administration of a F(ab’)_2_ polyclonal ovine antitoxin given 24 h post-exposure [69]. Even when performed in the same animal species, comparison between diverse experiments is often difficult, due to the different toxin dose and route of administration utilized. Moreover, there are few data about correlation between the antitoxin dose required for protection and the WOO. 

## 5. Ricin-Containing Immunotoxins

Many researchers have tried to exploit the high cytotoxicity of ricin for medical purposes to eliminate pathological cells. Although ricin possesses highly efficient cell killing mechanisms, it lacks selectivity towards cell targets. In order to increase selectivity, the possibility of linking ricin to carriers specific for targets on unwanted cells has been explored. The most widely used carriers are antibodies and the corresponding conjugates are referred to as immunotoxins (ITs).

The first IT, created in 1976 by Moolten and co-workers, was made by Ricin Toxin-A chain (RTA) linked to a rat tumor-specific antibody against a rat lymphoma, namely (C58NT)D (Figure 1) [107]. To date, a multitude of pre-clinical and clinical studies have shown the potential use of several ricin-ITs towards different cancer types, from hematological to solid ones, and towards normal cells, unwanted due to them being responsible for a pathological state (reviewed in [108,109]). Different approaches have been used, over time, to generate ITs. In the first strategy, ITs were composed by the antibody chemically linked to the entire RIP and they were used for in vitro studies showing high cytotoxicity [110]. Despite the high in vitro efficiency, the relevant non-specific toxicity reported in vivo, due to the characteristics of the lectin chain, brought researchers to sterically block, chemically modify, or remove the B chain, thus balancing toxicity and specificity. In 1985, Weil-Hillman et al. tested an anti-Mr 67,000 protein linked to either blocked-chain B ricin or RTA, reporting interesting results in vitro, but not in vivo in a nude mouse model [111]. The 1980s were years of great ferment for molecular biology and genetic engineering, paving the way for the second generation of ITs. Many researchers tried to improve the IT penetration in tumor mass by reducing the antibody size, using antigen-binding (Fab), or variable (Fv) fragments instead of entire antibodies. In 1988, Ghetie et al. created a new IT composed by Fab conjugated to chemically deglycosylated RTA (dgA) [112]. A few years later, they used an anti-CD122-dgA IT in SCID-Daudi mice, showing promising results since the IT was able to specifically kill tumor cells in vivo, extending the mean survival time up to 57.9 +/− 3.8 days [113]. Moreover, FitzGerald et al. described the antitumor activity of recombinant RTA (rRTA) linked to anti-mouse transferrin receptor in a nude mouse model of human ovarian cancer. Animals treated with IT had an extended life span from 45 (lower doses) to 70/80 days (higher doses) [114]. Finally, in 1997 the first ricin-containing recombinant immunotoxin (rIT) was obtained through the expression of a fusion gene composed by sequences encoding anti-CD19-FVS191 (single-chain Fv), cathepsin D proteinase digestion site, and rRTA. In this work, the authors compared the cytotoxicity of the rIT with the chemical linked IT, evidencing that only the latter was toxic in target cells [115]. About 20 ricin-ITs have been tested in Phase I, II, and III clinical trials to treat patients with tumors, either hematological or solid, transplant rejection, and GvHD. The first Phase I clinical trial, giving promising results, was conducted by Spitler et al. in 1987 (Figure 1), by treating 22 metastatic malignant melanoma patients with an IT composed by murine monoclonal anti-melanoma antibody coupled to RTA (XOMAZYME-MEL) [116,117,118]. Additionally, ITs were also exploited for the treatment of autoimmune diseases. Indeed, anti-CD5/RTA was the first IT used in clinical trials for therapy of autoimmune diseases, such as rheumatoid arthritis, systemic lupus erythematosus, and insulin-dependent diabetes mellitus [119,120]. The advantages and limitations of ricin containing ITs for cancer therapy were recently discussed together with strategies for reducing the immunogenicity of recombinant ITs [121,122].

A different new approach consists of nanoparticle construction, in which ricin is genetically fused to carrier peptides that are able not only to recognize specific cellular target, but also to auto assemble, as stable nanoparticles, thus increasing the toxin-concentration into the targeted site [109,123,124]. 

## 6. Conclusions

In conclusion, ricin is a highly cytotoxic plant protein and has been of great utility to develop a number of anti-cancer immunotoxins. Ricin, and of some other RIPs, are able to act on multiple molecular targets inside the cell, thus triggering different death pathways; this makes such proteins more attractive for cancer treatment than conventional chemotherapy, in which one of the major problems is the rise of resistant cells [67,125]. However, ricin-containing ITs have also been shown to exhibit many limitations, such as unspecific toxicity, organ toxicity (mainly liver, kidney, and vasculature), immunogenicity, fast removal from blood stream, and lysosomal degradation inside cells. As a result, despite of the significant efforts made over the past few years, ricin as therapeutic agent has not achieved much impact at the clinical level. The challenge is still open, and frontline research is directed towards recombinant immunotoxins and nanocarriers, or towards other novel techniques such as the vector-driven expression of active plant toxin genes in tumor cells [109,126]. 

Although ricin is not toxic enough to hypothesize a use over a wide area for terrorist purposes, the availability of castor beans and the quite simple procedure for rough ricin purification have stimulated the interest of criminals and terrorists for small-scale biocrimes. This justifies researchers’ efforts to obtain faster and more sensitive ricin detection tests. Furthermore, the study of inhibiting or neutralizing molecules and the timing of clinical events following ricin intoxication could lead to the definition of one or more validated therapies.

Finally, the use of castor bean derivatives should be carefully monitored because of the potential presence of active ricin. In fact, the large use of these products in agriculture, without an effective ricin inactivation, has already caused several cases of animal intoxication, and can be hazardous for human health.
